# Supplementary material for: Complex‐centric proteome profiling by SEC‐SWATH‐MS
Source: Mol Syst Biol. 2019 Jan 14;15(1):e8438. doi: 10.15252/msb.20188438 (PMC6346213; doi:10.15252/msb.20188438)
Supplement: Supplementary file 8 — Dataset EV7 [file MSB-15-e8438-s008.zip › feature_plots_string/Q96A35.pdf]

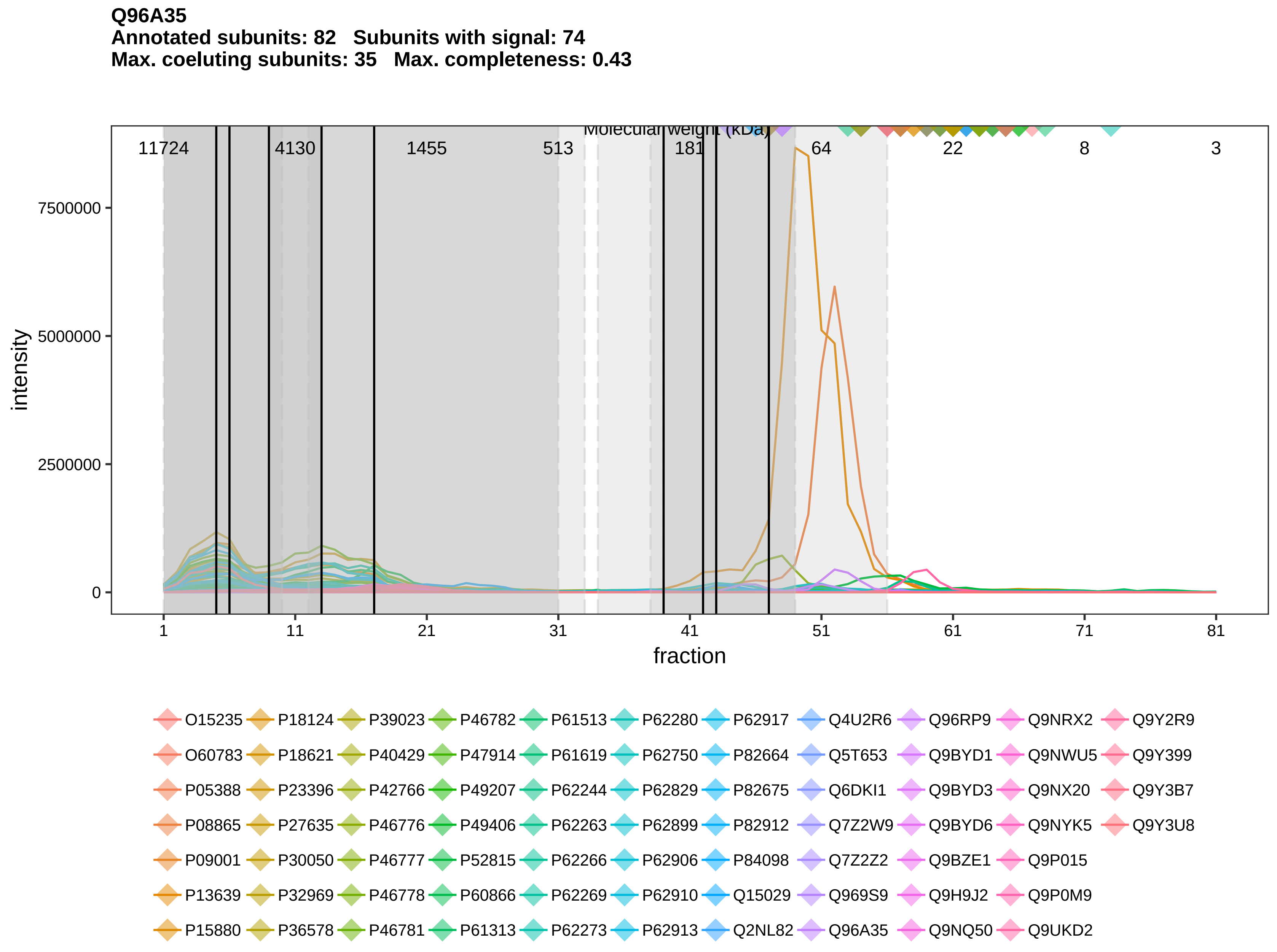
Q96A35
Annotated subunits: 82 Subunits with signal: 74
Max. coeluting subunits: 35 Max. completeness: 0.43
Molecular weight (kDa)
11724
4130
1455
513
181
64
22
8
3
7500000
5000000
2500000
0
intensity
1
11
21
31
41
51
61
71
81
fraction
O15235
P18124
P39023
P46782
P61513
P62280
P62917
Q4U2R6
Q96RP9
Q9NRX2
Q9Y2R9
O60783
P18621
P40429
P47914
P61619
P62750
P82664
Q5T653
Q9BYD1
Q9NWU5
Q9Y399
P05388
P23396
P42766
P49207
P62244
P62829
P82675
Q6DKI1
Q9BYD3
Q9NX20
Q9Y3B7
P08865
P27635
P46776
P49406
P62263
P62899
P82912
Q7Z2W9
Q9BYD6
Q9NYK5
Q9Y3U8
P09001
P30050
P46777
P52815
P62266
P62906
P84098
Q7Z2Z2
Q9BZE1
Q9P015
P13639
P32969
P46778
P60866
P62269
P62910
Q15029
Q969S9
Q9H9J2
Q9P0M9
P15880
P36578
P46781
P61313
P62273
P62913
Q2NL82
Q96A35
Q9NQ50
Q9UKD2
